# Treatment of Gastric Cancer Means Surgery, but Not Surgery Alone

**DOI:** 10.3390/cancers16081601

**Published:** 2024-04-22

**Authors:** Manrica Fabbi, Christina D. Bali, Georgios D. Lianos, Stefano Rausei

**Affiliations:** 1 Department of General Surgery, Cittiglio-Angera Hospital, ASST Settelaghi, 21033 Varese, Italy; fabbi.manrica@gmail.com; 2 Department of Surgery, University Hospital of Ioannina, 45332 Ioannina, Greece; cbali@uoi.gr (C.D.B.); georgiolianos@yahoo.gr (G.D.L.)

Despite numerous studies, gastric cancer (GC) still presents a high mortality rate in Eastern and Western countries, increasing attention for new therapeutic strategies. Even if surgery remains the cornerstone of GC management, a multimodal approach seems potentially able to improve overall survival [1].

From a surgical point of view, many topics and details are still debated, although the gold standard procedure is well defined (gastrectomy, total or subtotal, depending on the tumor side and its histology, with D2 lymphadenectomy). 

Given the strict correlation between the number of lymph nodes (LNs) harvested and survival rate [2,3], meticulous surgical technique and D2 LN dissection in locally advanced gastric cancer (LAGC) are the first steps for proper LN procurement, and the number of harvested LN is a direct measure of the quality of surgery [4]. The second (but not less important) step depends on the pathologic examination and identification of the LNs on the specimen, although success rates largely depend on the identification methods used [5,6], also considering that neoadjuvant chemotherapy alters the number of retrieved LN to a lower level [7]. 

Ambrosio et al. (contribution 1) have implemented a different approach to LN identification and examination following radical gastrectomy. Both the surgeon and pathologist were present in the operative theater and implicated in an on-site macroscopic evaluation and dissection of a fresh tissue specimen. The proposed protocol succeeded in identifying more LN in a non-time-consuming way through a surgeon–pathologist collaboration, even in patients receiving neoadjuvant therapy. In that way, proper staging was responsible for better overall treatment and survival. This study could be a precursor for other studies, necessary to confirm their findings and assess the impact of this technique on oncological outcomes. 

Another important aspect of GC surgical treatment is the omentectomy during radical gastrectomy. Total omentectomy (TO) has always been thought to be the standard surgical procedure for healing intent, as the omentum serves as a bridge for peritoneal metastasis [8]. On the other side, the omentum may participate in antibacterial defense, hemostasis and preventing intestinal adhesions [9]. Chai et al. (contribution 2) make an important contribution to this topic with their systematic review and meta-analysis investigating the safety and efficacy of partial omentectomy (PO) compared to TO. The study demonstrated the non-inferiority of PO in terms of long-term oncological outcomes, with better overall survival, shorter operative time, and lesser blood loss. Thus, TO could not be necessary to perform routinely.

Another aspect in which there is no univocal agreement is the technique used to perform the esophago-jejunal anastomosis after total gastrectomy. Charalabopoulos et al. (contribution 3) reported their experience in performing hand-sewn two-layer running suture esophago-jejunal anastomosis utilizing 3D vision in laparoscopic total gastrectomy. The post-operative anastomotic leak and stricture rates were negative, as were the 30- and 90-day mortality rates. A totally laparoscopic total gastrectomy requires precise surgical skills for complex anastomoses. Surgeons use stapled or hand-sewn techniques, with the latter demanding advanced laparoscopic suturing expertise. Robotic surgery may simplify hand-sewn anastomosis, addressing challenges in classic laparoscopic techniques. Despite advancements, esophagojejunal anastomotic leakage remains a serious complication with a reported incidence of 2.9–9%, associated with a poorer prognostic factor [10,11,12,13,14], prompting ongoing research to minimize risk given its significant impact on patient prognosis. 

Maintaining surgery, a fundamental role for potentially curative intent for early GC, it is now well established that LAGC should be treated with a multimodal approach. Perioperative chemotherapy, though standard worldwide, varies in drug regimens globally and within countries, even if 5-FU-based chemotherapy is currently the widely accepted backbone in the LAGC treatment. In Western countries, the FLOT regimen is the standard perioperative drug [15,16], replaced by FOLFOX or CAPOX in fragile patients, even if their well-known benefits are reduced [17,18]. On the contrary, S-1 is the first-line treatment for LAGC in Eastern countries, combined with cisplatin (SP) or oxaliplatin (SOX), with promising results [19,20,21,22,23]. Even if its efficacy in Caucasian populations has been previously demonstrated in randomized clinical trials [21,24,25]. The transferability of this regimen to the Western population is still under evaluation due to a different 5-FU metabolism between Asian and non-Asian people. In this still primordial framework, the retrospective study of Koumarianou et al. (contribution 4) reported real data about the use of S-1 combined with a platinum agent in the first-line setting of European patients with LAGC, demonstrating similar survival outcomes and toxicity profiles using this regimen with previously reported data from Asian populations. These promising data should be a stimulus to initiate randomized clinical trials in European populations to provide further insight into the evaluation of S-1 therapy in non-Asian patients. 

Perioperative chemotherapy seems not to be the only option for LAGC management, as the importance of integrated therapy has been addressed and new, experimental therapies targeting different molecular markers are continually updated. De Pascale et al. (contribution 5), evaluating the effects of the CROSS regimen (chemoradiotherapy followed by surgery) [26] in adenocarcinoma of the cardia, reported that chemoradiotherapy in the neoadjuvant setting seems to influence the site of recurrence, significantly reducing local recurrence.

A new promising target for anticancer therapy seems to be proangiogenic proteins that induce the proliferation, migration, invasion, and tube formation of endothelial cells, favoring tumor angiogenesis. While this type of target therapy is very popular in other types of cancer (in particular breast and prostate tumors), it is not yet well studied in GC. Despite some limitations of the study, Kalfon et al. (contribution 6) showed that angiopoietin-2 is expressed not only in primary GC but also in omental metastasis, suggesting that ANG2 may promote metastasis by stimulating angiogenesis in the omental metastatic niche. These data could be in favor of performing a total omentectomy while also keeping this topic open. Thus, further studies are needed to validate this interesting preliminary finding.

The microbiota of the stomach has long been suspected to have a role in gastric carcinogenesis, although limited progress has been made regarding the definite role of non-*H. pylori* in the development of GC. The review published by Pappas-Gogos et al. (contribution 7) provides an overview of the topic, highlighting the putative role of the non-*H. pylori* microbiome and their metabolites in enhancing the effects of some traditional antineoplastic drugs and immunotherapies. However, no explicit microbiota and their metabolites have been identified as a predominant indicator of GC development or their exact role in therapies. Therefore, further investigations are required to elucidate the detailed carcinogenic mechanisms of the gastric microbiome and provide novel insights for GC management.

Van Amelsfoort et al.’s (contribution 8) review emphasizes the importance of assessing health-related quality of life (HRQOL) in GC treatment. The study reported impaired HRQOL post-surgery or neoadjuvant/adjuvant therapy, regardless of surgery type. Similar patterns of low HRQOL occur post-chemoradiation. Although the authors admit lacking high-quality studies, the topic and results are of great relevance since they stress the importance of also focusing on the psychologic and physical consequences of the treatments offered to patients, not only on the disease. QOL scores should be included in patients records in order to extract more accurate conclusions about the incidence of impaired QOL and its implications for patients’ lives.

GC remains a pathology with a poor prognosis despite significant treatment advancements. Further research is essential to address challenges such as early detection, recurrence reduction, and treatment optimization. Moreover, GC recurrence remains common, prompting ongoing studies to identify high-risk individuals’s post-treatment. Anticipating treatment response is crucial, and molecular GC classification could provide novel pathways for patient stratification and targeted therapies. To fully understand the mechanism of resistance to therapy (chemo or immune), factors such as epigenetics, metabolism, immune suppression, and microbiota must be considered. Challenges persist in determining optimal treatment strategies and timing for molecular biomarker screening. Extensive translational research and multi-omics-based trials are expected to drive breakthroughs in GC diagnosis and treatment.

This Special Issue explores GC treatment advancements over the past two decades, focusing on molecular insights, innovative drugs, surgical advancements, technological innovations, and modern clinical approaches. Physicians are dedicated to improving patient outcomes, and future studies hold promise for enhancing clinical practice.

## Abbreviations

FLOT	fluorouracil + leucovorin + oxaliplatin + docetaxel
FOLFOX	folinic acid + fluorouracil + oxaliplatin
CAPOX	oxaliplatin + capecitabine
S-1	tegafur + gimeracil + oteracil
